# The impact of hospital wide measures to reduce mupirocin resistance among methicillin-resistant *Staphylococcus aureus* in a Singapore hospital

**DOI:** 10.1017/ash.2025.147

**Published:** 2025-09-03

**Authors:** HML Oh, J Chen

**Affiliations:** 1 Department of Infectious DiseasesChangi General Hospital, Singapore

## Abstract

**Introduction:** Methicillin-resistant Staphylococcus aureus (MRSA) is a leading cause of healthcare associated infections. Colonization with MRSA increases the risk of subsequent nosocomial infection. The primary concern regarding widespread use of mupirocin is the emergence of mupirocin resistance. A prospective cross-sectional study in Singapore in 2013, found mupirocin resistance to be 31.6% in Changi General Hospital (CGH). Annual usage of mupirocin (g) in CGH was 36870 and hospital-onset MRSA bacteremia was 1.1/10,000 patient-days in 2013. **Objective:** To study the impact of hospital measures to reduce mupirocin resistance among MRSA by detection of mupirocin resistance in screening isolates. **Method:** Changi General Hospital is a 1000 bedded acute care hospital. Hospital wide measures were instituted in CGH to reduce mupirocin resistance in MRSA included a) universal body wash with Octenidine for all hospitalized patients in the wards with MRSA cubicles b) 2% mupirocin ointment removed from formulary (available for nasal decolonization only) A study was conducted on MRSA screening isolates from the Microbiology Laboratory between May and September 2019. These were obtained by swabbing nasal, axilla and groin on all newly admitted patients as part of an active surveillance program since 2013. The swabs were streaked onto MRSA^ii^selective media plates which were incubated at 35 °C for 20 hours and stored at 4 °C. E-test was performed to determine the susceptibility and minimum inhibitory concentration (MIC) of MRSA isolates to mupirocin, oxacillin and vancomycin, following the CLSI guidelines for S. aureus. MPCR (multiplex polymerase chain reaction) assay was used for the simultaneous identification of *ileS*-2 (primers MupA and MupB) and mecA (primers MecA1 and MecA2). PCR amplification of *ileS*- 2 gene for high level mupirocin resistance and Mec A gene was performed on Touch thermal cycler. **Results:** 200 MRSA isolates were tested. E-test revealed 5 isolates were detected to be High Level mupirocin- resistant (2.5%) and 69 isolates were detected to be oxacillin-resistant (74%). The MPCR assay detected mecA gene in 100% and *ileS*-2 gene in 3 isolates (1.5%). **Conclusion:** Our study indicated the low prevalence of high level mupirocin resistance among MRSA screening isolates in 2019 in CGH. This suggested that the hospital wide measures to reduce mupirocin resistance were effective.

